# The complete chloroplast genome sequence of *Magnolia maudiae*

**DOI:** 10.1080/23802359.2020.1715894

**Published:** 2020-01-24

**Authors:** Yongkang Sima, Yunqing Li, Yi Wang

**Affiliations:** Laboratory of Forest Plant Cultivation and Utilization, Yunnan Academy of Forestry and The Key Laboratory of Rare and Endangered Forest Plants of State Forestry Administration, Kunming Yunnan, People’s Republic of China

**Keywords:** *Magnolia maudiae*, chloroplast, Illumina sequencing, phylogenetic analysis

## Abstract

The first complete chloroplast genome (cpDNA) sequence of *Magnolia maudiae* was determined from Illumina HiSeq pair-end sequencing data in this study. The cpDNA is 160,205 bp in length, contains a large single-copy region (LSC) of 88,249 bp and a small single-copy region (SSC) of 18,806 bp, which were separated by a pair of inverted repeats (IR) regions of 26,575 bp. The genome contains 132 genes, including 87 protein-coding genes, 8 ribosomal RNA genes, and 37 transfer RNA genes. Further phylogenomic analysis showed that *M. maudiae* was close to *Magnolia odora* and *Magnolia laevifolia* in *Magnolia* genus.

*Magnolia maudiae* (Dunn) Figlaris [synonymous with *Michelia maudiae* Dunn] is the species of the genus *Magnolia* within the family Magnoliaceae (Xiong and Liu 2018). *Magnolia maudiae* is a unique precious tree species in China, native in Zhejiang, Fujian, Hunan, Guangdong, Guangxi and Guizhou (Sun et al. 2011). Their timber is used for furniture, board, drawing board, and joinery. It also is an important ornamental plant in South China (Lang et al. 2019). The volatile oil of *M. maudiae* had antibacterial and antitumor properties (Cao et al. 2007). Therefore, *M. maudiae* has a huge value. However, there have been no genomic studies reported on *M. maudiae*.

Herein, we report and characterize the complete plastid genome of *M. maudiae*. The GenBank accession number is MN897727. One *M. maudiae* individual was collected from Kunming arboretum, Yunnan Academy of Forestry, Yunnan Province of China (25°14′18″N, 102°75′23″E). The specimen is stored at Yunnan Academy of Forestry Herbarium, Kunming, China, and the accession number is S96019. DNA was extracted from its fresh leaves using DNA Plantzol Reagent (Invitrogen, Carlsbad, CA, USA).

Paired-end reads were sequenced by using Illumina HiSeq system (Illumina, San Diego, CA). In total, about 8.4 million high-quality clean reads were generated with adaptors trimmed. Aligning, assembly, and annotation were conducted by CLC *de novo* assembler (CLC Bio, Aarhus, Denmark), BLAST, GeSeq (Tillich et al. 2017), and GENEIOUS v 11.0.5 (Biomatters Ltd, Auckland, New Zealand). To confirm the phylogenetic position of *M. maudiae*, other eight species of *Magnolia* genus from NCBI were aligned using MAFFT v.7 (Katoh and Standley 2013). The auto algorithm in the MAFFT alignment software was used to align the 10 complete genome sequences and the G-INS-i algorithm was used to align the partial complex sequences. The maximum likelihood (ML) bootstrap analysis was conducted using RAxML (Stamatakis 2006); bootstrap probability values were calculated from 1000 replicates. *Liriodendron tulipifera* (MK477550) and *Liriodendron chinense* (KU170538) were served as the out-group.

The complete *M. maudiae* plastid genome is a circular DNA molecule with the length of 160,205 bp, contains a large single-copy region (LSC) of 88,249 bp and a small single-copy region (SSC) of 18,806 bp, which were separated by a pair of inverted repeats (IR) regions of 26,575 bp. The overall GC content of the whole genome is 39.2%, and the corresponding values of the LSC, SSC, and IR regions are 37.9%, 34.2%, and 43.2%, respectively. The plastid genome contained 132 genes, including 87 protein-coding genes, 8 ribosomal RNA genes, and 37 transfer RNA genes. Phylogenetic analysis showed that *M. maudiae* was close to *Magnolia odora* and *Magnolia laevifolia* in *Magnolia* genus (Figure 1). Determination of the complete plastid genome sequences provided new molecular data to illuminate the *Magnolia* genus evolution.

**Figure 1.**
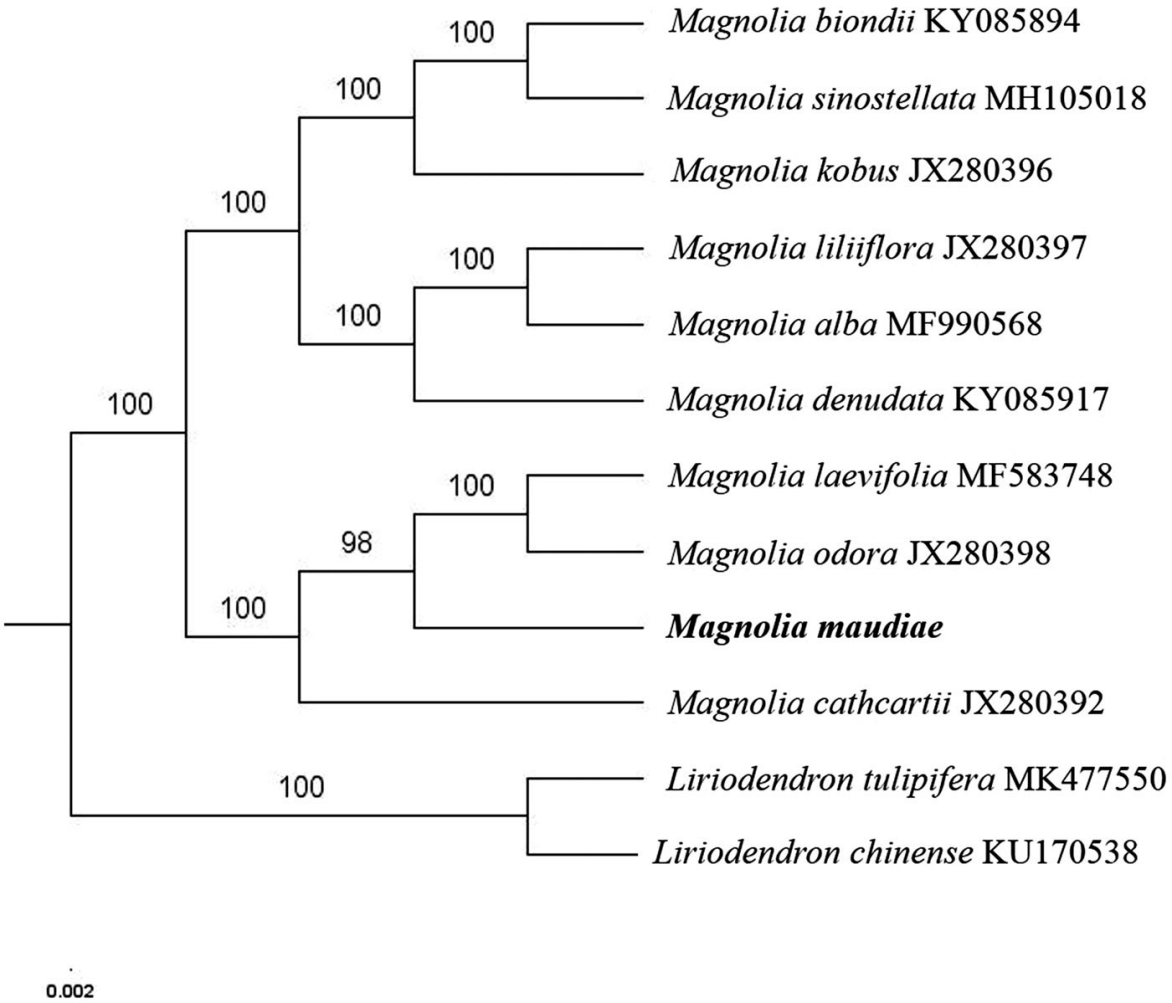
The maximum-likelihood tree based on the ten chloroplast genomes of *Magnolia* genus. The bootstrap value based on 1000 replicates is shown on each node.
